# The Eye of the Beholder: Attitudes toward divorced parents and perception of children's happiness in Peru and Spain

**DOI:** 10.1016/j.heliyon.2024.e36260

**Published:** 2024-08-14

**Authors:** Miguel Clemente, Pablo Espinosa, J. Alonso Aguilar-Valera, Claudia Guevara-Cordero

**Affiliations:** a Universidade da Coruna (Spain), Department of Psychology, Elvina's Campus, 15071 A Coruna, Spain; b National University of San Marcos, Department of Psychology, Lima, Peru; c Universidad Privada del Norte. Department of Psychology, Lima, Peru

**Keywords:** Child-parent-relations, Dark personality, Divorce, Extended family, Grandparent-grandchild relationships

## Abstract

Studies of divorce's effects on children have been oriented toward the parents' characteristics, ignoring their extended families. In the current study we collected data from 414 participants, both divorced parents and the children's extended families in Peru (155) and Spain (259). Participants completed a questionnaire on attitudes toward the parents, and the Short Dark Tetrad questionnaire. Multivariate tests were conducted on participants' responses, showing that negative attitudes toward parents are not very strong but that attitudes toward fathers are more negative than attitudes toward mothers. Custodial parents are perceived less negatively, and parents who share custody are perceived the least negatively. Extended family members hold more negative attitudes against parents than the parents themselves. Mothers have significantly more negative attitudes toward fathers than the fathers towards mothers. Dark traits are a significant predictor of negative attitudes toward the mother but not toward the father. The perception of unhappiness in the child was predicted by negative attitudes toward the parents. Non-parents tend to evaluate both parents more negatively compared to parents' mutual perceptions. There was an effect on dark traits, negative attitudes against parents, and perception of unhappiness in the child. Peruvians scored significantly higher in all variables.

## 1 Introduction

The purpose of this survey study is to examine how divorced parents and extended family perceive former spouses and how this influences the perception of children's well-being. The novelty of this research consists on examining the impact of custody and the perception of parents from the perspective of both divorced parents and extended family members.We use participant samples from two different countries to check whether these perceptions are stable across different cultural settings.

Perhaps the best-known document that recognizes children's defence is the Universal Declaration of the Rights of the Child [1], which specifies ten fundamental rights for children. None of them explicitly references happiness, but upon reading them, the substrate of all of them can be considered achieving happiness. But what happens when the family—normally comprising both parents of the child—breaks up? How does that affect the children's happiness?

When a couple with children in common breaks up, a series of beliefs are triggered [2]. These beliefs, which could be defined as myths, are as follows: the breakup of the parents’ relationship is negative for the child; divorce is a concrete milestone, a specific moment in time, not a process; this breakup implies that at least the contact between the child and one of the parents (the non-custodial parent) is clearly impaired, implying an emotional estrangement between the child and that parent; when the non-custodial parent has a new partner, the relations with their biological children worsen; if the custodial parent has a new partner, again, this creates conflict between the stepparent and the stepchild; and after the breakup, if there is a legal dispute over the custody of the children, this struggle is transferred to the families, which support the partner they have a kin-relationship with, and this also has a negative impact on children.

The nature of the relationship after divorce influences ex-partners well-being. The new situation may be experienced as more stressful and devoid of rules [2]. When there is joint physical custody, parents express more satisfaction with life than parents who have sole physical custody [3]. Parents who are in good terms with their former spouse and forgive offenses they may have held against them also score higher in life satisfaction [4].

As for children of divorced parents well-being, divorce is difficult for children to overcome, but it does not always have negative repercussions [5]. Gierveld and Merz [6] reported that many children struggle to keep their family together, both initially from their biological families and subsequently from their reconstituted families [7]. In that sense, Aquino, Brand, and Torche [8] stated that whereas some children were strongly affected by the breakup, others experienced few consequences. The impact of divorce on children is influenced by variables like socioeconomic resources or ethnicity [9]. However, although for some children divorce may not be traumatic, its effects are predominantly negative [10]. As regards the new situation after divorce, The parenting of divorced fathers and mothers is equally important for children, so joint custody is in the best interest of the child. The support and parenting of both parents increase the well-being of children [11]. Nevertheless, the importance of post-divorce parenting reflects the pre-divorce involvement of the parent in child-rearing [12]. Pre-separation couple relationships are related to involvement of fathers after divorce. Prior mother's perception of father's competence is positively linked to the level of contact that fathers maintain [13]. Conversely, perceptual divergence with father's rearing skills is a predictor of conflict between the ex-couple [14]. Joint custody increases children's well-being [[15], [16], [17]]. This effect is mediated by the quality of family relationships [18]. When custody is not shared, research [19] has shown that children experiment more well-being in the custody of their father, as they often benefit from maintaining a positive relationship with their mother. The bottom line is that children's happiness is largely explained by positive social interactions involving family and friends [20].

Extended family plays an important role in the divorce process and also suffers the consequences of the family break up. For instance, divorce often increases the levels of hostility of divorcees, which may influence interpersonal relationships both with the ex-partner and extended family [21]. At any rate, divorce alters parents' relationship with extended family. After divorce, parents reduce contact with spouse's extended family and receive more support from their own family [22]. However, contact with and support obtained from former in-laws may continue depending on the quality of the relationship before divorce [23]. Maintaining contact with and receiving support from other family members after divorce is associated to higher well-being [24]. In the case of grandparents, they usually change their customary role [25]. These changes are explained by matrilineal advantage theory [26], which states that paternal grandparents are more at risk of having little or no contact with their grandchildren. Jappens and Van Bavel [25] state that, although relationships with maternal grandparents tend to be closer than with paternal grandparents, the strength of the relationships with both maternal and paternal grandparents is positively associated with the well-being of grandchildren with divorced parents. This relationship with their grandparents is maintained through time, supporting the kin-keeper theory [27]. A determining issue in the future contact of children of divorced parents with their grandparents is the judicial court arrangements [28]. Thus, grandchildren of divorced parents have fewer contacts with their grandparents than grandchildren of married parents. However, when the grandchildren of divorced parents live with their father or in a joint custody arrangement, they see their paternal grandparents even more often than do grandchildren of married parents. Contact with their grandparents provides children with emotional and practical support [29].

Another concern in our study is that perceptions of parents may be biased by the respondent characteristics. Personality variables may influence other people perceptions. In this study we focus on a subset of personality characteristics, the dark traits of personality, that are related to self-centeredness, antagonism and negative outcomes for others [30,31]. Dark traits are related to externalizing biases or to punishing and blaming other people [32]. Dark traits are also related to dichotomous thinking or seeing the world in black or white [33]. This bias may lead to all-or-nothing thinking and judging others as good or bad instead of considering possible nuances. Psychopathy has also been found to be related less agreeableness and hostility towards others [34]. For this reason, we believe that it is relevant to consider the influence of dark traits in people's evaluations of divorced parents.

## 2 Objectives and hypotheses

The main objective of this study is to examine how parental perception influences perceived child happiness and how custody arrangements influence both parent's perception and children perceived happiness. Since parental involvement is considered beneficial for the child [11] we expect that custodial parents will be perceived more positively, and that happiness of the child will be perceived as higher when there is joint custody [[15], [16], [17]]. Additionally, we expect that biases related to dark traits of personality (Jonason et al., 2009, 2018) will influence the perception of divorced parents. Since these findings from previous research have been tested in different countries and settings, we expect that results will be similar for both of our participant samples. We examine children of divorced parents perceptions of happiness in a novel way, combining attitudes, perceptions and personal characteristics of both parents and extended family across two different samples. Thus, we propose the following hypotheses.

- H1: Attitudes toward the custodial parent will be less negative than attitudes toward the non-custodial parent.
- H2: Attitudes toward the parents will positively predict participants' perceptions of child well-being.
- H3: The perception of the child's well-being will be more positive when parents have joint custody.
- H4: High scores on dark personality traits will predict negative attitudes toward the parents, due to the antagonistic nature of these traits. Our hypothesis is that the influence of dark traits on interpersonal attitudes may act as a bias that distorts the participant's perception of the parents.
- H5: Participants' attitudes toward parents and their perceptions of child well-being will vary. Compared to parents, other respondents (e.g., non-parents) will have more negative attitudes and perceptions. Our hypothesis is that being less familiar with the divorce situation will lead to a more negative evaluation.
- H6: The explanatory variables of participants' attitudes toward the parents and their perception of child well-being will be stable in all settings. Our hypothesis is that the same model will explain these variables in the samples of both Peru and Spain.

## 3 Method

### 3.1 Participants

Participants were divorced parents or their relatives from Peru and Spain, who were asked about their perception of the parents and the child involved in the divorce. Thus, they were selected because they had children and had been involved in a divorce or because they had first-hand knowledge of a situation of this kind. Originally, 553 participants, 298 Spanish and 255 Peruvian, responded to the study. However, 139 had to be excluded from the study for failing consistency checks (127), answering about their own experience as children of divorced parents (5), being underage, or failing to state their age (7). The final sample comprised 414 participants, 155 from Peru and 259 from Spain. There was an attrition rate of 13.1 % in the Spanish sample, and 39.2 % in the Peruvian sample.

Peruvian participants’ mean age was 31.46 (*SD* = 11.96); 56 % were female. All participants were Peruvian nationals.

Participants from Spain were 40.67 years old on average (*SD* = 18.07), and over two-thirds (69.7 %) were female. Most participants in this sample were Spanish (89.6 %).

### 3.2 Measures

The participants indicated their relationship with the child involved, the child's age, and the person in charge of the child. They also completed a questionnaire about their attitudes toward both the father and the mother (10 items for each) and about how the child experienced the situation (7 items) on a 5-point scale ranging from 1 (*strongly disagree*) to 5 (*strongly agree*). Sample items were: “The child does not love him/her,” “He/she does not know how to educate the child,” “The child is not happy,” or “I feel guilty for not being able to help the child more.” Thus, high scores indicated negative attitudes toward the parents or a negative perception of the child's well-being. Reliability scores for the attitudes toward parents were calculated, yielding very acceptable results both for the father's (α = .93), and the mother's scale (α = .90). For this study, we selected the belief that the child was not happy as a measure of the respondent's perception of the child.

Participants also answered the Short Dark Tetrad (SD4) questionnaire [35]. This 28-item, 5-point Likert scale, which ranges from 1 (*strongly disagree*) to 5 (*strongly agree*), measures the dark traits that comprise the dark tetrad, namely Machiavellianism (manipulative and calculating attitudes to achieve personal goals at the expense of others), Narcissism (feeling superior to others as well as dominating behaviors and seeking admiration from others), Psychopathy (lack of empathy toward others and impulsiveness), and Sadism (enjoying provoking or watching harm in other people). The reliability scores for the four scales in this study were acceptable (Machiavellianism: α = .78, Narcissism: α = .87, Psychopathy: α = .86, and Sadism: α = .83).

### 3.3 Procedure

Data for the study was collected through a survey, which is a common technique in the research on this topic [16] Participants were contacted by university students who collaborated in exchange for course credit. The criteria for participation were being the parent or being acquainted with a child whose parents had undergone a divorce. Eligible participants were provided with a link to an online survey. Before responding to the questionnaire, they read an informed consent statement about the tasks they were to perform in the study and its voluntary and confidential nature. They also received the means to contact researchers if they had further questions or wanted to receive the results from the study after its completion. Response time was about 20 min on average. Participants were required to answer every item. The study did not allow missing responses.

To avoid careless or inattentive responses, we followed one of the procedures recommended by Maniaci and Rogge [36] by including two items where we asked participants to respond, marking a given number. If participants failed to respond correctly to either item, we excluded them from the study.

Before performing the investigation, permission was requested from the Research Center Ethics Committee of the corresponding author. The research meets the ethical criteria of the Helsinki Protocol and the American Psychological Association.

### 3.4 Data analysis

We initially conducted a correlational analysis to check how participants' attitudes toward parents were related to their perception of the child. In these correlations, we controlled for the participants’ sex.

Paired-sample *t*-tests were performed to compare attitudes toward fathers and mothers within the same participant, and additional *t*-tests were calculated to compare participants by sex, parent versus non-parent respondents, and by sample origin. An analysis of variance (*ANOVA*) was also conducted to compare attitudes toward parents and perception of the child as a function of who the child lived with. Furthermore, a multivariate covariance analysis (*MANCOVA*) was conducted to explain how the participant's relationship with the child and the parent with whom the child lived influenced attitudes toward the father, the mother, and perceptions of the child, using the participant's dark traits as a covariate.

Finally, we developed SEM models to check how the person with whom the child lived, attitudes toward parents, and dark traits predicted the perceptions of the child, comparing participants’ samples and parents versus non-parents.

## 4 Results

Sample characteristics for Peruvian and Spanish participants differed significantly in gender (χ^2^(1) = 7.65, *p* = .008), age (χ^2^(63) = 92.94, *p* = .008), educational level (χ^2^(4) = 26.62, *p* = .001), religion (χ^2^(6) = 72.72, *p* = .001) and relation with the child (χ^2^(3) = 18.23, *p* = .001). Table 1 shows the differences in education religion adscription and relation with the child across samples.

**Table 1:** Descriptive statistics for Peruvian and Spanish participants.

	Peru	Spain
Education		
Primary	15,10 %	5,20 %
Secondary	34,40 %	49,70 %
Vodational training	15,10 %	23,90 %
Bachelor degree	26,60 %	18,70 %
Master degree	8,90 %	2,60 %
Religion		
Catholic	70,30 %	50,60 %
Agnostic	12,30 %	13,50 %
Atheist	1,30 %	31,30 %
Other	16,10 %	3,10 %
Relation with child		
Parent	22,70 %	27,09 %
Grandparent	14,67 %	3,22 %
Uncle	35,52 %	48,39 %
Other	27,02 %	21,29 %

Participants responded to several questions about the child involved in the divorce under consideration. In the Peruvian sample, participants indicated that the child was on average 8.71 years old on average (*SD* = 5.39). Most children lived only with the mother (58.7 %), although 27.7 % lived with both parents, and 7.1 % lived only with the father. The remaining 4.5 % lived with other relatives. Over half the children (54.2 %) had no siblings. In the sample from Spain, the reference child was 10.69 years old on average (*SD* = 4.74) and usually lived with the mother (45.2 %) or both parents (45.6 %). Only 6.6 % lived only with the father, and 2.3 % lived with other people. Most children (59.5 %) were only children.

### 4.1 Correlational analysis

Correlational analyses were conducted to explore the relationship between the study variables. To avoid gender biases against the mother or the father, the sex of the participant was controlled for in these correlations.

The belief that the child was not happy was related to negative attitudes toward the father (*r* = .51, *p* < .001) and the mother (*r* = .48, *p* < .001). Dark traits were related to negative attitudes toward the mother (Machiavellianism *r* = .21, *p* < .01; narcissism: *r* = .24, *p* < .01; psychopathy: *r* = .28, *p* < .001; sadism: *r* = .22, *p* < .01), but not to negative attitudes toward the father or perceptions of the child. Psychopathy predicted the strongest attitudes toward the mother.

### 4.2 *T*-tests and ANOVAs

Each participant informed about their attitudes toward the child's father and mother. To compare responses from the same participant about the mother and the father, paired-sample *t*-tests were conducted. They showed significant differences in individual attitudes toward the father and the mother, except for physical aggression. As we calculated a general scale comprising all the individual attitude items, the paired-sample *t*-test for this scale summarizes these results, revealing significant differences (*t*[413] = 9.23, *p* < .001; *r* = .27, *p* < .001). This test shows that, on average, participants had worse attitudes toward the father (*M* = 1.87, *SD* = .98) than toward the mother (*M* = 1.41, *SD* = .62).

*T*-tests comparing results by participant's sex only yielded differences in psychopathy (*t*[412] = 3.48, *p* < .001; males: *M* = 1.83, *SD* = .78; females: *M* = 1.57, *SD* = .67) and sadism (*t*[412] = 6.08, *p* < .001; males: *M* = 2.18, *SD* = .91; females: *M* = 1.68, *SD* = .73). In both cases, male participants scored higher than female participants. No other differences were found in participants' attitudes toward the parents or their perception of the child.

We also compared participants who were the child's parents with other participants. *T*-tests showed slight differences in psychopathy (*t*[412] = 2.96, *p* < .01; parents: *M* = 1.86, *SD* = .86; non-parents: *M* = 1.61, *SD* = .67). However, there were significant differences in attitudes toward the father (*t*[412] = 2.92, *p* < .01; parents: *M* = 1.62, *SD* = .82; non-parents: *M* = 1.94, *SD* = 1.01), toward the mother (*t*(412) = 2.63, *p* < .01; parents: *M* = 1.27, *SD* = .35; non-parents: *M* = 1.45, *SD* = .68), and the perception that the child was not happy (*t*(412) = 2.45, *p* < .05; parents: *M* = 1.70, *SD* = 1.15; non-parents: *M* = 2.04, *SD* = 1.22). Results showed that both groups (parents vs. non-parents) scored very low on the scales. Parents scored slightly higher in psychopathy than non-parents. Interestingly, non-parents had significantly worse opinions of both parents and believed that the child was not happy more strongly than the parents themselves.

Considering only parents, there was a significant difference in negative attitudes toward the father, showing that mothers had a worse perception of fathers compared to the fathers’ own perception (*t* [99] = 2.61, *p* < .05: fathers: *M* = 1.35, *SD* = .54; mothers: *M* = 1.78, *SD* = .91). However, there were no differences between parents in attitudes toward the mothers. As for comparisons with the non-parents, there were no gender differences in attitudes toward fathers or mothers.

We conducted a final *t*-test to compare participants according to the sample's origin (Peru vs. Spain). Results show differences in most variables of the study. Table 1 shows that the Peruvian sample scored higher in all the dark traits except for sadism and showed significantly higher negative attitudes toward both parents and a stronger perception that the child was unhappy.

To check whether living with either parent influenced attitudes toward the parents or the perception of the child's happiness, we performed an ANOVA (Table 2), dividing the sample according to who the child lived with. Broadly, there were four possible custody situations: either living with only the mother or only the father, living with both parents, or other situations (e.g., living with grandparents). Post-hoc comparisons using Scheffe's test showed differences for all three variables depending on the custody situation.

**Table 2:** T-test by sample origin.

	*t* (412)	Spain *M* (*SD*)	Peru *M* (*SD*)
Machiavellianism	2.87^b^	2.57 (.92)	2.82 (.73)
Narcissism	12.12^c^	2.11 (.82)	3.11 (.89)
Psychopathy	5.50^c^	1.52 (.58)	1.91 (.87)
Negative attitudes toward father	5.96^c^	1.65 (.83)	2.22 (1.10)
Negative attitudes toward mother	5.92^b^	1.27 (.48)	1.63 (.76)
Belief that the child is not happy	2.56^a^	1.84 (1.20)	2.15 (1.22)

a *p* < .05.

b *p* < .01.

c *p* < .001.

Attitudes toward the father were more negative when the child did not live with him (lived with the mother or another situation). Similarly, when the child lived with the father but not with the mother, attitudes toward the mother were more negative. The child was perceived as less happy when living only with the mother compared to living with both parents. Thus, being the custodial parent reduced participants’ negative attitudes, and the child was perceived as happier when living with both parents (Table 3).

**Table 3:** ANOVA by custody situation.

	*F* (3, 406)	Mother *M* (*SD*)	Father *M* (*SD*)	Both *M* (*SD*)	Other *M* (*SD*)
Negative attitudes toward father	28.58^b^	2.24 (1.10)_a,b_	1.37 (.34)_a,c_	1.43 (.59)_b,d_	2.32 (1.17)_c,d_
Negative attitudes toward mother	5.80^a^	1.41 (.64)_a_	1.76 (.76)_a,b_	1.32 (.53)_b_	1.78 (.71)
Belief that the child is not happy	5.79^a^	2.17 (1.28)_a_	1.89 (1.03)	1.66 (1.07)_a_	2.31 (1.55)

Note: Means sharing subscripts differ significantly at *p* < .05.

a *p* < .01.

b *p* < .001.

All *ANOVA* models were significant. There was a main effect for psychopathy as a predictor of negative attitudes toward mothers (*r* = .29, *p* < .001) and fathers (*r* = .15, *p* < .01), but psychopathy had no main effect on the perception of the child's happiness (Table 4).

**Table 4:** MANCOVA model.

		*df*	*F*	η^2^
Negative attitudes toward father	Corrected Model	7, 413	16.24^c^	.22
	Parent vs. non-parent by gender	3, 413	4.74^b^	.03
	Father x Mother custody	3, 413	26.15^c^	.16
	Psychopathy	1, 413	15.49^c^	.04
Negative attitudes toward mother	Corrected Model	7, 413	10.56^c^	.15
	Parent vs. non-parent by gender	3, 413	5.30^c^	.04
	Father custody x Mother custody	3, 413	5.35^c^	.04
	Psychopathy	1, 413	49.39^c^	.11
Belief that the child is not happy	Corrected Model	7, 413	3.74^c^	.06
	Parent vs. non-parent by gender	3, 413	2.78^a^	.02
	Father custody x Mother custody	3, 413	5.26^c^	.04
	Psychopathy	1, 413	.71	.002

a *p* < .05.

b *p* < .01.

c *p* < .001.

Parent versus non-parent status, taking participants' sex into account, also had a small main effect on the criteria variables (η^2^ ranging from .02 to .16). Post-hoc comparisons using Scheffe's test only revealed significant differences for negative attitudes toward fathers. In this case, as Table 5 indicates, the non-parent participants had significantly more negative attitudes toward the parents than the parents themselves did. Post-Hoc comparisons for the other two variables showed similar patterns, although not significant when taken individually.

**Table 5:** Main Effect of Gender Combined with Parent vs. Non-parent Status. Post-hoc Comparisons.

	Father *M* (*SD*)	Mother *M* (*SD*)	Other Male *M* (*SD*)	Other Female *M* (*SD*)
Negative attitudes toward father	1.35 (.54)_a,b_	1.78 (.91)	1.89 (1.07)_a_	1.98 (.98)_b_
Negative attitudes toward mother	1.32 (.38)	1.24 (.33)	1.44 (.66)	1.46 (.69)
Belief that the child is not happy	1.65 (.98)	1.73 (1.25)	1.90 (1.19)	2.14 (1.23)

*Note*: Means sharing subscripts differ significantly at *p* < .05.

We found a small interaction effect size for custody (η^2^ = .04). Custody indicated whether the child lived with the father or the mother. A child living with both parents was included in both variables in the “yes” category.

**Fig. 1:**
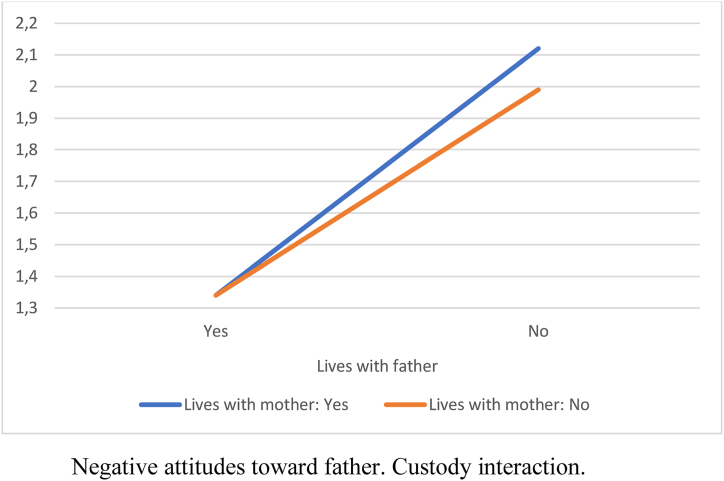
Negative attitudes towards father.Custody interaction.

**Fig. 2:**
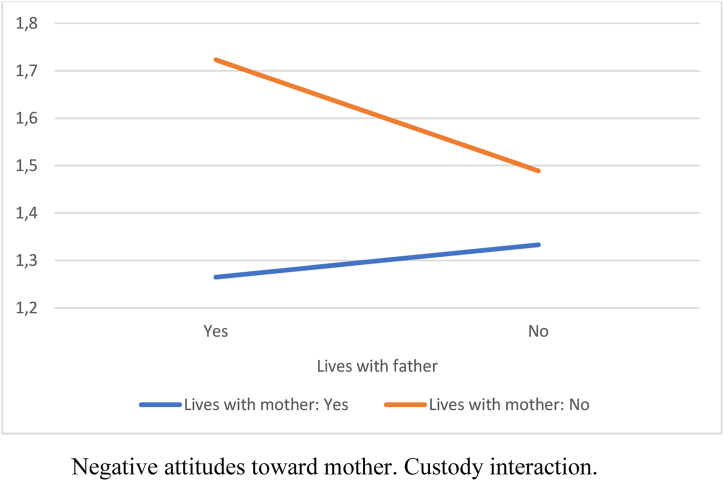
Negative attitudes towards mother.Custody interaction.

**Fig. 3:**
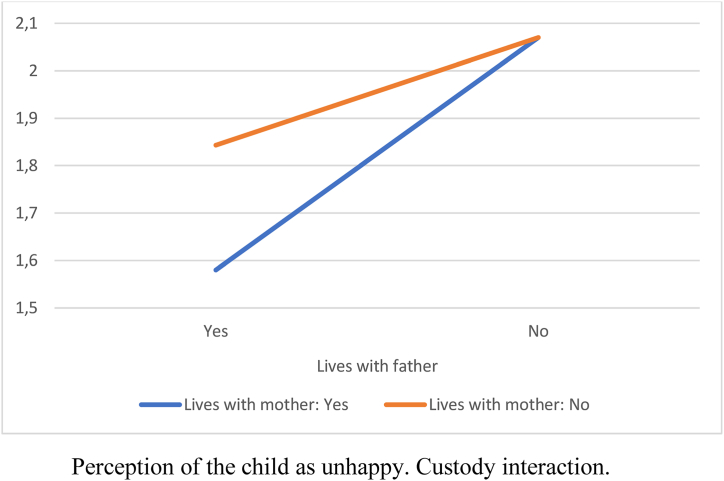
Perception of the child as unhappy.Custody interaction.

Fig. 1, Fig. 2, Fig. 3 show the interaction effects. In every instance, not living with the mother increased negative attitudes toward her and the perception of the child's unhappiness. When the child did not live with either the father or the mother, these negative attitudes and the perception of the child's unhappiness were further increased. The least negative attitudes and the highest perception of the child's happiness occurred when the child lived with both parents.

### 4.3 SEM models

We developed an explanatory SEM model to determine how the perception of the child's happiness was influenced by negative attitudes against the parents. We used custody status as a predictor for attitudes toward the parents and included psychopathy to check whether participants' bias may also have been a significant predictor.

**Fig. 4:**
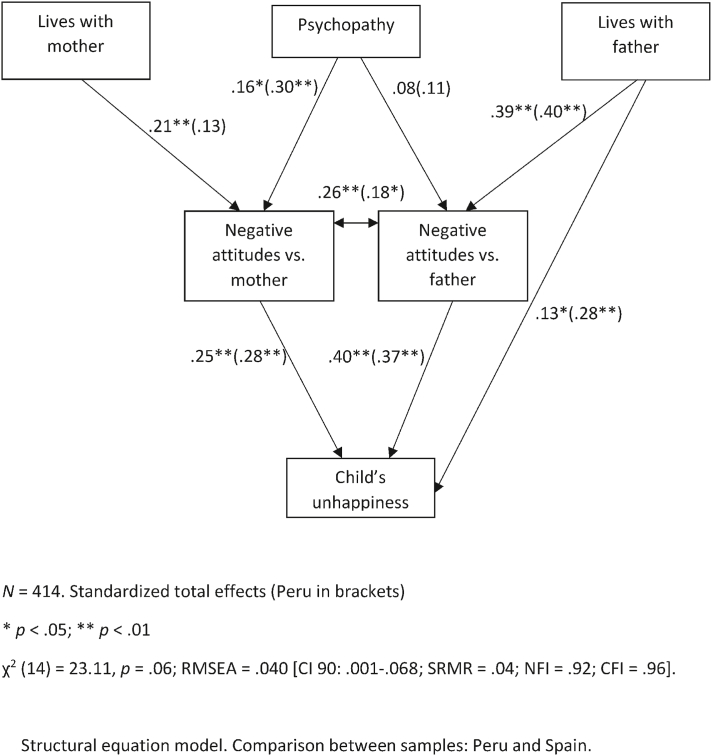
Structural equation model.Comparison between samples:Peru and Spain.

Fig. 4 below shows that the SEM model was significant, and all the goodness-of-fit indexes were adequate. Z-scores of critical ratios for differences between parameters showed no differences in the relationship between variables across samples. There were no mediation effects in this model, but in the Spanish sample, we found several indirect effects between living with the father and the perception of the child's unhappiness (*r* = .15, *p* < .01), living with the mother and the perception of the child's happiness (*r* = .05, *p* < .01), and psychopathy and the perception of the child's unhappiness (*r* = .07, *p* < .05). In the Peruvian sample there were indirect effects of living with the father and the child's perceived unhappiness (*r* = .15, *p* < .01), and psychopathy (*r* = .12, *p* < .01), but not for living with the mother.

Overall, this model showed good explanatory power for both samples, whose parameters did not differ as indicated by the critical ratios. Living with either parent was coded as 1 = yes, 2 = no, so positive parameters in this model mean that not living with the father or the mother increased negative attitudes toward him or her. Psychopathy biased participants' attitudes toward the parents and their perceptions of the child, whereas living with the father (either only with him, which was infrequent, or with both parents) predicted the child's perceived happiness. Not surprisingly, having negative attitudes toward either parent predicted perceiving the child's unhappiness.

**Fig. 5:**
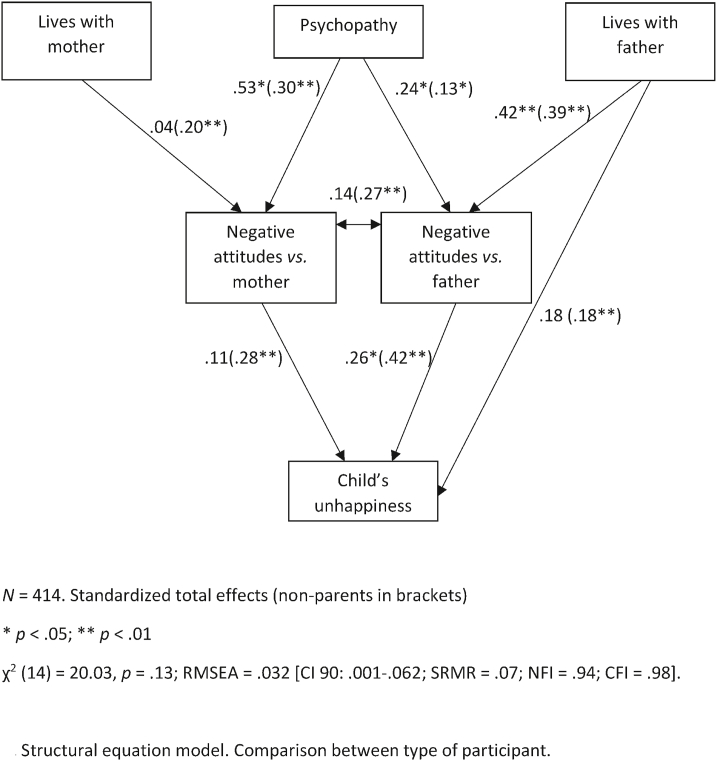
Structural equation model.Comparison between type of participant.

Finally, we ran the same SEM model, this time comparing types of participants. For this analysis, we divided the sample between parents and non-parents (Fig. 5).

This model also had a good fit, showing that among parents, mothers' variables neither predicted negative attitudes toward them nor the perception of the child's unhappiness. However, among non-parents, mothers' indicators were significant predictors. Living with the father did not predict the perception of the child's unhappiness, probably because of the smaller sample size in the parent group.

Among the parents, negative attitudes toward fathers were unrelated to attitudes toward mothers (as shown in Table 4, mothers had stronger negative attitudes toward fathers, but not the opposite). However, among non-parents, both attitudes were very significantly correlated. Critical ratios showed significant differences between these two parameters (*z* = 3.85, *p* < .01). Also, critical ratios indicated another significant difference between parameters in the relationship between living with the mother and attitudes toward her (*z* = 2.61, *p* < .05). The rest of parameters follow the same pattern as in Fig. 4. There were no mediation effects in the parent group, but there was an indirect effect of living with the father on the perception of the child's unhappiness (*r* = .11, *p* < .05). In the non-parent group, there were indirect effects for living with the father (*r* = .40, *p* < .01), living with the mother (*r* = .22, *p* < .01), and psychopathy (*r* = .25, *p* < .01) in the perception of the child's unhappiness.

## 5 Discussion and conclusions

Our results show that the perception of children's happiness in divorced couples depends on the custody status of the child and on the attitudes towards parents, which are subject to interpersonal biases. These results highlight the importance of joint custody and how attitudes towards parents shape the perception of the child well-being. Participants' perception on custody is aligned with previous research on custody and well-being [[15], [16], [17]].

Children of divorced parents are not perceived as unhappy in general, so the belief that divorce is negative for the child [2] is not shared by our participants. There is a number of determinants (custody, perception of parents, respondent characteristics) that influence the perception of whether the child is happy or not. However, the belief that the divorce results in the strangement of at least one of the parents may be implied in the importance given to custody and the more favorable attitudes towards custodial parents. In this sense, our first hypotheses is supported by the fact that attitudes towards custodial parents are better than attitudes towards non-custodial parents. This perception is consistent with previous research [11] underlining the importance of continued parenting. Most children of divorced parents live either with the mother o with both parents. Only a minority live with their father only. A positive perception of parents increases the perceived child's happiness, as we expected in out second hypothesis. This is also consistent with previous research on the quality of family relations [18]. The parent with custody of the child is perceived less negatively, but additionally parents who share custody are perceived the least negatively. The least negative attitudes and best perception of the child's happiness occurred when there was joint custody. This result supports our third hypothesis and is in line with extant literature [[15], [16], [17]]. In this vein, Holder and Coleman [20] argued that the variation in children's happiness is largely explained by positive social interactions involving family and friends.Conversely, when the child lives with people who are not their parents, the negative attitudes against parents are increased. Living with the father and not with the mother significantly increases the negative attitudes towards the mother. This is probably because it is an uncommon or unexpected situation, given that most children live with their mothers alone or in shared custody.

As predicted in our fourth hypothesis, high levels of dark traits in repondents are related to more negative attitudes towards parents. Dark traits explain negative attitudes towards parents. Dark traits may lead to biased thinking when appraising parents and children in a divorce situation. Psychopathy is the strongest predictor of negative attitudes, although it is unrelated with perceptions of the child unhappiness. This is congruent with antagonistic tendencies in people high in psychopathic traits [32,33].

As regards out fifth hypothesis, non-parents (extended family) have more negative attitudes toward the parents than the parents about themselves. Within the parents, the mothers have significantly more negative attitudes toward the fathers than the fathers about themselves. However, the fathers do not evaluate the mothers more negatively. In general, negative attitudes towards parents are not very high, but attitudes towards fathers are more negative than attitudes towards mothers. - There were no gender differences in attitudes towards parents or the perception of the child.

Finally, our sixth hypotheses predicted that attitudes and perceptions will be similar across settings. Our SEM model comparing samples revealed no significant differences across participant samples. The samples were not only from different countries, but they were not equal in composition (age, gender, education, religion).Additionally, there was s a sample effect on dark traits, negative attitudes against parents and perception of unhappiness in the child. Peruvians score significantly higher in all these variables. However, there were no significant differences in our explanatory model, which is an indication that the relationship between variables is robust and stable across settings.

The SEM models show that the perception of unhappiness in the child is predicted by negative attitudes toward the parents. In turn, these attitudes are predicted by personality traits (psychopathic tendencies) in the respondent, which can lead to biased attitudes, Personality traits and custodial status indirectly predict perceived unhappiness in the child. This model adequately explains the attitudes and perceptions both in the Spanish and Peruvian samples, so it is sufficiently robust to be used with other samples, although the Peruvian sample scored higher in all the study variables. This is consistent with Steinbach and Hank [37] findings about cultural variations. In summary, our results provide support for all hypotheses in the study.

These results show that the conflicts derived from divorce also influence extended families but does not cause extreme attitudes and that the child well-being is evaluated condidering parents perceived competence and the new custody situation. This is in contradictios with the tenets of the PAS theory established by Gardner and agrees with its critics [[38], [39], [40], [41], [42]].

On the other hand, the data show that respondents evaluate mothers more positively and perceive the mother's parenting as highly important, and evaluate mothers more negatively when they do not hold the child's custody.This supports the matrilinear advantage theory [26], as well as the kin-keeper theory [27].

This research has a number of limitations: the sample size, which is small; inequality across samples, and the uneven type of situations evaluated, as we could count on few families in which the fathers had custody (although this fact reflects the reality of the judges' decisions on the children's custody). Comparison across differen extendend family roles (i.e. Grandparents *vs* uncles; kin *vs*. in-laws) could not be evaluated due to sample size. The small number of variables studied is also a limitation, considering that for future work, for example, the variable network of children's friends should also be included. However, we think that one of the strengths of this work is to obtain quantitative and psychological data on the extended family in relation to the parents' divorce and the children's happiness.

The results of this research show how extended families attenuate the conflict that divorce can produce between parents and allow us to identify the type of personality that characterizes relatives and close people who inappropriately appraise how parents socialize their children. It highlights the importance of joint custody and that attitudes towards parents, as an indicator of their perceived competence, shape the perception of the child well-being. It is also worth noting that perceptions are subjective and influence by the respondent personality. Findings in this study may provide insight to monitor the weel-being of children after divorce, and to consider the potential biases that former spouses or extended family witnesses muy incur in reporting parenting issues. Further research should explore whether the quality of parenting and joint custody may hinder or enhance the happiness of the child.

## 6 Ethics declarations

This study was reviewed and approved by ECRIM (Criminoloxía, Psicoloxía Xurídica e Xustiza Penal at Universidade da Coruña) research ethics committee with the approval number: 21-004, dated September 09, 2021.

All participants provided written informed consent to participate in the study and for the use of their data in research and publication. Data collection was anonymous.

## Funding

This research was supported by GRC grant ED431C 2023/24 from 10.13039/501100010801Xunta de Galicia, Spain.

## Data availability statement

Data are available at dx.doi.org/10.6084/m9.figshare.23537073.

## CRediT authorship contribution statement

**Miguel Clemente:** Writing – review & editing, Writing – original draft, Visualization, Validation, Supervision, Software, Resources, Project administration, Methodology, Investigation, Funding acquisition, Formal analysis, Data curation, Conceptualization. **Pablo Espinosa:** Writing – original draft, Visualization, Validation, Supervision, Software, Resources, Methodology, Investigation, Formal analysis, Data curation, Conceptualization. **J. Alonso Aguilar-Valera:** Writing – review & editing, Writing – original draft, Visualization, Validation, Supervision, Software, Methodology, Conceptualization. **Claudia Guevara-Cordero:** Writing – review & editing, Writing – original draft, Visualization, Validation, Supervision, Software.

## Declaration of competing interest

The authors declare the following financial interests/personal relationships which may be considered as potential competing interests:Miguel Clemente reports financial support was provided by 10.13039/501100010801Xunta de Galicia. If there are other authors, they declare that they have no known competing financial interests or personal relationships that could have appeared to influence the work reported in this paper.

## References

[1] United Nations A. (1989). General, convention on the rights of the child, 20 november 1989, annu. Rev. Popul. Law.

[2] Kalmijn M. (2016). Children's divorce and parent-child contact: a within-family analysis of older European parents, J. Gerontol. Series B.

[3] Augustijn L. (2023). Post-separation care arrangements and parents' life satisfaction: can the quality of Co-parenting and frequency of interparental conflict explain the relationship?. J. Happiness Stud..

[4] Yárnoz-Yaben S., Garmendia A., Comino P. (2016). Looking at the bright side: forgiveness and subjective well-being in divorced Spanish parents. J. Happiness Stud..

[5] Kalmijn M. (2015). How childhood circumstances moderate the long-term impact of divorce on father-child relationships. J. Marriage Fam..

[6] Gierveld J.D., Merz E.M. (2013). Parents' partnership decision making after divorce or widowhood: the role of (Step)Children. J. Marriage Fam..

[7] Connidis I.A. (2015). Exploring ambivalence in family ties: progress and prospects. J. Marriage Fam..

[8] Aquino T., Brand J.E., Torche F. (2022). Unequal effects of disruptive events. Sociol. Compass.

[9] D'Onofrio B., Emery R. (2019). Parental divorce or separation and children's mental health. World Psychiatr..

[10] Nambiar P.P., Jangam K.V., Jose A., Seshadri S.P. (2022). Predictors of behavioral and emotional issues in children involved in custody disputes: a cross sectional study in urban Bengaluru. Asian J Psychiatr.

[11] Bastaits K., Mortelmans D. (2014). Does the parenting of divorced mothers and fathers affect children's well-being in the same way?, child indic. Res..

[12] Poortman A.R. (2018). Postdivorce parent-child contact and child well-being: the importance of predivorce parental involvement. J. Marriage Fam..

[13] Petren R.E., Ferraro A.J., Pinto E. (2024). Pre-Separation family relationships and post-separation involvement among nonresident fathers in the United States. J. Fam. Stud..

[14] Madden-Derdich D.A., Leonard S.A. (2002). Shared experiences, unique realities: formerly married mothers' and fathers' perceptions of parenting and custody after divorce. Fam. Relat..

[15] Sodermans A.K., Matthijs K. (2014). Joint physical custody and adolescents' subjective well-being: a personality X environment interaction. J. Fam. Psychol..

[16] Steinbach A. (2019). Children's and parents' well-being in joint physical custody: a literature review. Fam. Process.

[17] Bjarnason T., Bendtsen P., Arnarsson A.M., Borup I., Iannotti R.J., Löfstedt P., Haapasalo I., Niclasen B. (2012). Life satisfaction among children in different family structures: a comparative study of 36 western societies, child. Soc.

[18] Steinbach A., Augustijn L. (2022). Children's well-being in sole and joint physical custody families. Fam. Psychol.

[19] ClarkeStewart K.A., Hayward C. (1996). Advantages of father custody and contact for the psychological well-being of school-age children. J. Appl. Dev. Psychol..

[20] Holder M.D., Coleman B. (2009). The contribution of social relationships to children's happiness. J. Happiness Stud..

[21] Overup C.S., Cipric A., Kjeld S.G., Strizzi J.M., Sander S., Lange T., Hald G.M. (2020). Cooperation after divorce: a randomized controlled trial of an online divorce intervention on hostility. Psychol. Violence.

[22] Gürmen M.S., Anderson S.R., Brown E. (2021). Relationship with extended family following divorce: a closer look at contemporary times. J. Fam. Stud..

[23] Duranaydintug C. (1993). Relationships with former in-laws - normative guidelines and actual behavior. J. Divorce & Remarriage.

[24] de Bel V. (2023). Multi-functional ties and well-being in family networks before and after parental divorce. Soc. Sci..

[25] Jappens M., Van Bavel J. (2020). Grandparent-grandchild relationships and grandchildren's well-being after parental divorce in Flanders, Belgium. Does lineage matter?. Jfr-J. Fam. Res..

[26] Sims M., Rofail M. (2013). The experiences of grandparents who have limited or no contact with their grandchildren. J. Aging Stud..

[27] Attar-Schwartz S., Fuller-Thomson E. (2017). Adolescents' closeness to paternal grandmothers in the face of parents' divorce. Child. Youth Serv. Rev..

[28] Jappens M., Van Bavel J. (2016). Parental divorce, residence arrangements, and contact between grandchildren and grandparents. J. Marriage Fam..

[29] Moore E., Timonen V., O'Dwyer C., Doyle M. (2012). Divorce and intergenerational support: comparing the perceptions of divorced adults and their parents. J. Comp. Fam. Stud..

[30] Moshagen M., Hilbig B.E., Zettler I. (2018). The dark core of personality. Psychol. Rev..

[31] Moshagen M., Zettler I., Hilbig B.E. (2020). Measuring the dark core of personality. Psychol. Assess..

[32] Jonason P.K., Fletcher S.A., Hashmani T. (2019). Externalizing and internalizing, but not rationalizing: some psychodynamic biases associated with the Dark Triad traits. Pers. Individ. Differ..

[33] Jonason P.K., Oshio A., Shimotsukasa T., Mieda T., Csathó A., Sitnikova M. (2018). Seeing the world in black or white: the Dark Triad traits and dichotomous thinking. Pers. Individ. Differ..

[34] Decuyper M., De Pauw S., De Fruyt F., De Bolle M., De Clercq B.J. (2009). A meta-analysis of psychopathy-, antisocial PD- and FFM associations. Eur. J. Pers..

[35] Paulhus D.L., Buckels E.E., Trapnell P.D., Jones D.N. (2021). Screening for dark personalities the Short dark tetrad (SD4). Eur. J. Psychol. Assess..

[36] Maniaci M.R., Rogge R.D. (2014). Caring about carelessness: participant inattention and its effects on research. J. Res. Pers..

[37] Steinbach A., Hank K. (2016). Intergenerational relations in older stepfamilies: a comparison of France, Germany, and Russia. J. Gerontol. Series B.

[38] Clemente M., Espinosa P. (2021). Revenge in couple relationships and their relation to the dark triad. Int. J. Environ. Res. Publ. Health.

[39] Clemente M., Espinosa P., Padilla D. (2019). Moral disengagement and willingness to behave unethically against ex-partner in a child custody dispute. PLoS One.

[40] Clemente M., Padilla-Racero D., Espinosa P. (2019). Revenge among parents who have broken up their relationship through family law courts: its dimensions and measurement proposal. Int. J. Environ. Res. Publ. Health.

[41] Clemente M., Padilla-Racero D., Espinosa P. (2020). The dark triad and the detection of parental judicial manipulators. Development of a judicial manipulation scale. Int. J. Environ. Res. Publ. Health.

[42] Clemente M., Padilla-Racero D., Espinosa P., Reig-Botella A., Gandoy-Crego M. (2019). Institutional violence against users of the family law courts and the legal harassment scale. Front. Psychol..

