# Supplementary material for: The Eye of the Beholder: Attitudes toward divorced parents and perception of children's happiness in Peru and Spain
Source: Heliyon. 2024 Aug 14;10(16):e36260. doi: 10.1016/j.heliyon.2024.e36260 (PMC11378926; doi:10.1016/j.heliyon.2024.e36260)
Supplement: Multimedia component 1 [file mmc1.pdf]

Departamento de Psicología
UNIVERSIDADE DA CORUÑA

English Translation of the questionaire

- **The original questionnaire, in Spanish, can be consulted at the end of this document.**
- **The standardized questionnaires SD-4, PMD, as well as the sociodemographic questions, do not appear in this English translation and can be consulted in Spanish.**

QUESTIONNAIRE ON RELATIONSHIPS OF CHILDREN WITH GRANDPARENTS, UNCLES AND OTHER RELATIVES AFTER A BREAKUP OF THE PARENTS' RELATIONSHIP

Good morning/afternoon: A team of professors from the Department of Psychology at the University of La Coruña (Spain) is conducting research on the behavior of the extended family (grandparents and uncles especially) with children, when the parents have bullied their children. relationship (they have been divorced), for which it is necessary to have the valuable help of people who complete the following questionnaire.

In this sense, we ask you to collaborate with us by answering the following questions spontaneously and keeping in mind that we only want to know your answer to the questions posed. Do not leave any item unanswered, as this would invalidate the questionnaire.

To be able to participate, it is essential that you have a relationship with a child between 0 and 18 years old who is either your grandson or your nephew, or is a child of your current partner and that said relationship is continuous (have contact with him or her at least once a month).

The information you provide us will be treated in a general way along with that of other participants, guaranteeing anonymity and being used exclusively for scientific research purposes. If you have any questions about the questionnaire, or the research in which it is part, you can contact Professor Miguel Clemente (miguel.clemente@udc.es).

PART 1 – SOCIODEMOGRAPHIC DATA

Translator's note: The sociodemographic data was introduced next.

11. **Next we are going to ask you some questions regarding the children's fathers, the mothers, the children and you. Respond according to the scale from "strongly agree" to "strongly disagree."**

1	2	3	4	5
Completamente en desacuerdo	En desacuerdo	Ni de acuerdo ni en desacuerdo	De acuerdo	Completamente de acuerdo
Completely disagree	Disagreement	Neither agree nor disagree	Agree	Completely agree

**Questions about the children's father:**					
The children do not love this parent	1	2	3	4	5
He doesn't know how to educate his children	1	2	3	4	5
He may physically attack his children	1	2	3	4	5
He Psychologically abuses his children	1	2	3	4	5
He does not take proper care of his children	1	2	3	4	5
He does not contribute money for the care of his children, if it is his obligation	1	2	3	4	5
It is rare that he gives gifts to his children	1	2	3	4	5
It is rare that he calls his children when he is not with them.	1	2	3	4	5
I believe that he/she does not love his/her children.	1	2	3	4	5
I think he uses his children to go against the other parent	1	2	3	4	5
**Questions about the children's mother:**					
The children do not love this parent	1	2	3	4	5
She doesn't know how to educate his children	1	2	3	4	5
She may physically attack his children	1	2	3	4	5
She Psychologically abuses his children	1	2	3	4	5
She does not take proper care of his children	1	2	3	4	5
She does not contribute money for the care of his children, if it is his obligation	1	2	3	4	5
It is rare that She gives gifts to his children	1	2	3	4	5
It is rare that She calls his children when She is not with them.	1	2	3	4	5
I believe that he/she does not love his/her children.	1	2	3	4	5
I think She uses his children to go against the other parent	1	2	3	4	5
**Questions about the child or children**					
I think the child is not happy	1	2	3	4	5
I think the child is being sexually abused	1	2	3	4	5
Since I am not the father or mother of the child, I do not feel capable of guiding him or her.	1	2	3	4	5

Since I am not the father or mother of the child, I do not feel capable of imposing punishments on him or her.	1	2	3	4	5
**Questions about you**					
Since I am not the father or mother of the child, I do not feel capable of guiding him/her.	1	2	3	4	5
Since I am not the child's father or mother, I do not feel empowered to impose punishments.	1	2	3	4	5
I think I love the child more than his parents.	1	2	3	4	5
I would be capable of breaking the law to defend the child.	1	2	3	4	5
I feel guilty for not being able to do more for the child.	1	2	3	4	5

SD4. Translator's note: The SD-4 questionnaire was introduced next.

PMD. Translator's note: The PMD questionnaire was introduced next.

THE QUESTIONNAIRE HAS FINISHED.

PLEASE CHECK THAT YOU HAVE NOT LEFT ANSWERS BLANK.

IF YOU WOULD LIKE TO MAKE ANY COMMENTS OR OBSERVATIONS, YOU CAN USE THE FOLLOWING SPACE:

THANK YOU VERY MUCH FOR YOUR HELP!

CUESTIONARIO SOBRE RELACIONES DE LOS NIÑOS CON ABUELOS, TÍOS Y OTROS FAMILIARES TRAS UNA RUPTURA DE LA RELACIÓN DE LOS PADRES

Buenos días / tardes: Un equipo de profesores del Departamento de Psicología de la Universidad de La Coruña (España) estamos realizando una investigación sobre el comportamiento de la familia extensa (abuelos y tíos sobre todo) con los niños, cuando los padres han toro su relación (se han divorciado) , para lo que es preciso contar con la valiosa ayuda de personas que cumplimenten el siguiente cuestionario.

En este sentido, le pedimos que colabore con nosotros respondiendo a las siguientes cuestiones de forma espontánea y teniendo en cuenta que solamente queremos saber su respuesta a las preguntas planteadas. **No deje ningún ítem sin responder**, ya que eso invalidaría el cuestionario.

Para poder participar es **imprescindible que usted tenga relación con un niño de entre 0 y 18 años que sea o su nieto o su sobrino, o sea un hijo/a de su actual pareja y que dicha relación sea continua** (tenga contacto con él al menos una vez al mes).

La información que nos aporte será tratada de forma general junto a la de otros participantes, **garantizando el anonimato y utilizándose exclusivamente con fines de investigación científica**. Si tiene alguna consulta sobre el cuestionario, o la investigación en la que se enmarca, puede contactar con el profesor Miguel Clemente (miguel.clemente@udc.es).

DATOS DE REGISTRO DEL CUESTIONARIO

Fecha de cumplimentación: ______ / ______ /______
ID Participante[1]:
Nombre del entrevistador:

[1]Indique los 4 últimos dígitos del DNI sin letra (para garantizar el anonimato).

PARTE 1 – DATOS SOCIODEMOGRÁFICOS

1. Sexo: ❑Hombre ❑ Mujer

2. Edad: __________

3. Nivel de formación (indique el más alto que posea):

❑ 1 Primaria/EGB ❑ 2 Secundaria/Bachiller ❑ 3 FP Grado Medio

❑ 4FP Grado Superior ❑ 5 Grado/Diplomatura/Licenciatura universitaria

❑6 Postgrado/Máster ❑ 7 Doctorado

4. Indique qué relación le une con el menor o los menores de 0 a 18 años:

❑ 1 Soy su padre/madre

❑ 2 Soy su abuelo/a

❑ 3 Soy su tío/ tía

❑ 4 Soy la pareja de su actual madre o padre

5. Indique su religión o si es usted ateo o agnóstico

1. ❑ Agnóstico
2. ❑ Ateo
3. ❑ Budista
4. ❑ Católico
5. ❑ Cristiano ortodoxo
6. ❑ Musulmán
7. ❑ Protestante
8. ❑ Otros (Indicar): ________________

6**. ¿Cuál es su nacionalidad?**

❑ 1 Rusa

❑ 2 Española

❑ 3 Otra (Indicar): ________________

7. **Indique si la relación es con un solo niño o si éste tiene hermanos, señalando las edades de cada uno. Si fueran más de seis señale solo los seis primeros:**

Niño más pequeño:	Edad: ____
Segundo niño más pequeño:	Edad: ____
Tercer niño más pequeño:	Edad: ____
4º	Edad: ____
5º	Edad: ____
6º	Edad: ____

8. ¿Tiene usted hijos (Indique cero o el número de los que tenga) ____

9. ¿Cuántos de ellos son menores de 18 años? (Indique cero o el número de los que tenga) _____

10. ¿Con quién convive habitualmente el niño?

- ❑ Con el padre
- ❑ Con la madre
- ❑ Con ambos por igual
- ❑ Con otras personas (indicar): _______

11. **A continuación le vamos a efectuar unas preguntas referentes a los padres de los niños, a las madres, a los niños y a usted. Responda según la escala desde "muy de acuerdo" a "muy en desacuerdo".**

1	2	3	4	5
Completamente en desacuerdo	En desacuerdo	Ni de acuerdo ni en desacuerdo	De acuerdo	Completamente de acuerdo

**Preguntas sobre el padre de los niños:**					
1. Sus hijos no le quieren	1	2	3	4	5
2. No sabe educarles	1	2	3	4	5
3. Es posible que les agreda físicamente	1	2	3	4	5
4. Les maltrata psicológicamente	1	2	3	4	5
5. No les cuida adecuadamente	1	2	3	4	5
6. No aporta dinero para su cuidado, si es su obligación	1	2	3	4	5
7. Es raro que les haga regalos	1	2	3	4	5
8. Es raro que les llame telefónicamente cuando no está con ellos	1	2	3	4	5
9. Creo que no quiere a sus hijos	1	2	3	4	5
10. Creo que utiliza a sus hijos para ir contra el otro progenitor	1	2	3	4	5
**Preguntas sobre la madre de los niños:**					
1. Sus hijos no le quieren	1	2	3	4	5
2. No sabe educarles	1	2	3	4	5
3. Es posible que les agreda físicamente	1	2	3	4	5
4. Les maltrata psicológicamente	1	2	3	4	5
5. No les cuida adecuadamente	1	2	3	4	5
6. No aporta dinero para su cuidado, si es su obligación	1	2	3	4	5
7. Es raro que les haga regalos	1	2	3	4	5
8. Es raro que les llame telefónicamente cuando no está con ellos	1	2	3	4	5
9. Creo que no quiere a sus hijos	1	2	3	4	5
10. Creo que utiliza a sus hijos para ir contra el otro progenitor	1	2	3	4	5
**Preguntas sobre el niño o los niños**					
1. Creo que el niño/a no es feliz	1	2	3	4	5
2. Creo que el niño sufre abusos sexuales	1	2	3	4	5
3. Cono no soy el padre o madre del niño/a, no me siento con capacidad para orientarle.	1	2	3	4	5
4. Cono no soy el padre o madre del niño, no me siento con capacidad para imponerle castigos	1	2	3	4	5
**Preguntas sobre usted**					
1. Cono no soy el padre o madre del niño/a, no me siento con capacidad para orientarle.	1	2	3	4	5
2. Como no soy el padre o madre del niño, no me siento con capacidad para imponerle castigos	1	2	3	4	5
3. Creo que quiero más al niño que sus padres	1	2	3	4	5
4. Sería capaz de saltarme la ley para defender al niño/a	1	2	3	4	5
5. Me siento culpable por no poder hacer más por el niño/a	1	2	3	4	5

SD4. Indique su grado de acuerdo con cada una de las siguientes afirmaciones. Para ello tenga en cuenta la siguiente valoración:

1	2	3	4	5
Completamente en desacuerdo	En desacuerdo	Ni de acuerdo ni en desacuerdo	De acuerdo	Completamente de acuerdo

1. No es prudente/inteligente dejar que la gente conozca tus secretos.	1	2	3	4	5
2. Debes tener a gente importante de tu parte, cueste lo que cueste.	1	2	3	4	5
3. Evita los conflictos directos con los demás porque pueden serte útiles en el futuro.	1	2	3	4	5
4. Mantén un perfil bajo si quieres salirte con la tuya.	1	2	3	4	5
5. Manipular la situación requiere planificación.	1	2	3	4	5
6. Hacer halagos en una buena manera de conseguir que la gente se ponga de tu lado.	1	2	3	4	5
7. Me encanta cuando un plan tramposo tiene éxito.	1	2	3	4	5
8. La gente me ve como un líder natural.	1	2	3	4	5
9. Tengo un talento único para persuadir a la gente.	1	2	3	4	5
10. Las actividades grupales suelen ser aburridas sin mí.	1	2	3	4	5
11. Sé que soy especial porque la gente me lo dice continuamente.	1	2	3	4	5
12. Tengo algunas cualidades excepcionales.	1	2	3	4	5
13. Es probable que acabe siendo una estrella en algún ámbito.	1	2	3	4	5
14. Me gusta lucirme de vez en cuando.	1	2	3	4	5
15. La gente suele decir que estoy fuera de control.	1	2	3	4	5
16. Tiendo a ir en contra de las autoridades y sus reglas.	1	2	3	4	5
17. He estado en más peleas que la mayoría de la gente de mi edad y sexo.	1	2	3	4	5
18. Tiendo a lanzarme primero y hacer preguntas después.	1	2	3	4	5
19. He tenido problemas con la ley.	1	2	3	4	5
20. A veces me meto en situaciones peligrosas.	1	2	3	4	5
21. La gente que se mete conmigo siempre se arrepiente.	1	2	3	4	5
22. Ver una pelea a puñetazos me emociona/excita.	1	2	3	4	5
23. Realmente disfruto con películas y videojuegos violentos.	1	2	3	4	5
24. Es divertido cuando gente idiota se cae de bruces.	1	2	3	4	5
25. Disfruto viendo deportes violentos.	1	2	3	4	5
26. Algunas personas merecen sufrir.	1	2	3	4	5
27. He dicho cosas malas en las redes sociales solo por diversión.	1	2	3	4	5
28. Sé cómo herir a alguien sólo con palabras.	1	2	3	4	5

PMD. Nos gustaría saber el grado de acuerdo o desacuerdo en el que se encuentra con respecto a las siguientes afirmaciones. Por favor, indique la puntuación correspondiente en la escala que se presenta a continuación:

1	2	3	4	5	6	7
Totalmente en desacuerdo	Bastante en desacuerdo	En desacuerdo	Ni de acuerdo ni en desacuerdo	De acuerdo	Bastante de acuerdo	Totalmente de acuerdo

1. Está justificado contar chismes de otros si eso protege a las personas que te importan.	1	2	3	4	5	6	7
2. Coger algo sin el permiso del dueño no está mal mientras solamente lo hayas cogido prestado.	1	2	3	4	5	6	7
3. Teniendo en cuenta como la gente exagera sobre ellos mismos, no es ningún pecado “inflar” un poco nuestro currículo.	1	2	3	4	5	6	7
4. Las personas no deberían considerarse responsables de hacer cosas incorrectas cuando simplemente hacen lo que les dice una autoridad.	1	2	3	4	5	6	7
5. No se puede culpar a la gente de cosas que están técnicamente mal si todos sus amigos hacen lo mismo.	1	2	3	4	5	6	7
6. Decir que se te han ocurrido a ti ideas que son de otros no es para tanto.	1	2	3	4	5	6	7
7. Algunas personas tienen que ser tratadas duramente porque no tienen sentimientos que puedan ser heridos.	1	2	3	4	5	6	7
8. La gente que es maltratada normalmente hace cosas para merecérselo.	1	2	3	4	5	6	7
9. Rodee con un círculo la opción “Bastante en desacuerdo”.	1	2	3	4	5	6	7

HA FINALIZADO EL CUESTIONARIO.

POR FAVOR, COMPRUEBE QUE NO HA DEJADO RESPUESTAS EN BLANCO.

SI DESEA REALIZARNOS ALGÚN COMENTARIO U OBSERVACIÓN, PUEDE UTILIZAR EL SIGUIENTE ESPACIO:

¡MUCHAS GRACIAS POR SU COLABORACIÓN!
